# Effects and Tolerance of Silymarin (Milk Thistle) in Chronic Hepatitis C Virus Infection Patients: A Meta-Analysis of Randomized Controlled Trials

**DOI:** 10.1155/2014/941085

**Published:** 2014-08-27

**Authors:** Zongguo Yang, Liping Zhuang, Yunfei Lu, Qingnian Xu, Xiaorong Chen

**Affiliations:** ^1^Shanghai Public Health Clinical Center Affiliated to Fudan University, No. 2901, Caolang Road, Jinshan District, Shanghai 201508, China; ^2^Key Laboratory of Infectious Diseases of State Administration of Traditional Chinese Medicine (Clinical Base), Shanghai 201508, China; ^3^Fudan University Shanghai Cancer Center, Shanghai 200032, China; ^4^Shanghai Medical College, Fudan University, Shanghai 200032, China

## Abstract

*Objective*. This study aimed to evaluate the efficacy and safety of silymarin on chronic hepatitis C virus- (HCV-) infected patients. *Methods*. Randomized controlled trials (RCTs) of silymarin in chronic HCV-infected patients up to April 1, 2014 were systematically identified in PubMed, Ovid, Web of Science, and Cochrane Library databases. *Results.* A total of 222 and 167 patients in five RCTs were randomly treated with silymarin (or intravenous silibinin) and placebo, respectively. Serum HCV RNA relatively decreased in patients treated with silymarin compared with those administered with placebo, but no significance was found (*P* = 0.09). Meta-analysis of patients orally treated with silymarin indicated that the changes of HCV RNA are similar in the two groups (*P* = 0.19). The effect on alanine aminotransferase (ALT) of oral silymarin is not different from that of placebo (*P* = 0.45). Improvements in quality-of-life (Short Form-36) in both silymarin and placebo recipients were impressive but relatively identical (*P* = 0.09). *Conclusion*. Silymarin is well tolerated in chronic HCV-infected patients. However, no evidence of salutary effects of oral silymarin has yet been reported based on intermediate endpoints (ALT and HCV RNA) in this population. Moreover, intravenous administration of silymarin should be further studied.

## 1. Introduction

Approximately 40% of patients with chronic hepatitis C virus (HCV) infection have reported the use of at least one herbal product [1, 2]. Silymarin, which is the collective name of flavonolignans (silybin or silibinin, silydianin, and silychristin) extracted from milk thistle, is one of the most frequently reported herbal remedies representing 72% of all herbals used by patients with HCV [1]. Furthermore, silymarin is the preferred herbal medicine for HCV [3]. In the last decades, approximately 12,000 papers were published regarding milk thistle used as antioxidant or chemopreventive and anticancer agent, particularly as hepatoprotectant. Although numerous studies have been conducted, no well-established evidence has been obtained yet regarding the recommended use of these substances in clinical practice [4].

Experimental and clinical studies have shown that silymarin exhibits pharmacological activities that can benefit patients with liver diseases. Although the active mechanism is incompletely understood, silymarin exhibits antioxidant, immunomodulatory, antifibrotic, antiproliferative, and antiviral activities [5–8]. However, studies have shown controversial conflicts concerning the effects of silymarin on chronic HCV infection. The clinical efficacy of silymarin in chronic HCV infection has not yet been demonstrated because of inconsistent results [3]. Although silymarin is highly absorbed after this drug is orally ingested and elicits a strong first-pass effect on the liver [7], strictly designed randomized controlled trials (RCTs) have shown that oral silymarin slightly affects liver function and HCV viral load [9–11]. Conversely, intravenous silymarin is well tolerated and elicits a substantial antiviral effect against HCV in nonresponders and during peritransplantation period [12–14].

In a meta-analysis on milk thistle for patients with liver diseases, no significant reduction in mortality or improvement in liver histology and function has been demonstrated, but data are very limited in which substantial beneficial or harmful effects of milk thistle on mortality are excluded [15, 16]. In another meta-analysis, milk thistle exhibits significant effects on several outcomes, such as liver-related mortality, but data remain inconclusive [17]. Rambaldi et al. [18] questioned the beneficial effects of milk thistle on patients with chronic HCV infection and highlighted the lack of high-quality evidence to support this intervention according to a systematic review. Rambaldi et al. [18] emphasized that RCTs on milk thistle versus placebo should adequately be conducted and reported.

Insufficient evidence supports or refutes recommending silymarin to treat patients with chronic hepatitis C [15, 16]. The therapeutic benefits of silymarin to patients with chronic HCV infection have not yet been well studied or established [3]. We summarized RCT data to probe the beneficial and harmful effects of silymarin on chronic HCV infection, thereby presenting clinical evidence for both physicians and patients.

## 2. Methods

### 2.1. Search Strategy

We searched PubMed, Ovid, Web of Science, and Cochrane Library databases until April 1, 2014. The following medical subject headings were used: “hepatitis C;” “silymarin;” “milk thistle;” “*Silybum*;” “silibinin;” “silybin;” “silydianin;” and “silychristin.” Electronic searches were supplemented with manual searches of reference lists used in all of the retrieved review articles, primary studies, and abstracts from meetings to identify other studies not found in the electronic searches. The literature was searched by two authors (ZG Yang and YF Lu) independently.

### 2.2. Study Selection

Two authors independently selected trials and discussed them with each other when inconsistencies were found. Articles that satisfy the following criteria were included: (1) for study types, RCTs; (2) for participants, patients with chronic HCV infection were randomly divided into two groups; (3) for interventions, silymarin or other types of milk thistle compared with placebo; (4) for outcome measures, serum HCV RNA titers, serum alanine aminotransferase (ALT) level, and/or Short Form- (SF-) 36 for quality of life; and (5) available full texts. Studies that included patients with liver diseases (e.g., HBV infection, human immunodeficiency virus infection, and hepatocellular carcinoma) other than HCV were excluded.

### 2.3. Data Extraction and Quality Assessment

Two researchers independently read the full texts and extracted the following contents: publication data; study design; sample size; patient characteristics; treatment protocol; and outcome measures. The methodological qualities of the included RCTs were assessed according to Cochrane Collaboration's Tool described in Handbook version 5.1.0 [19]. Two authors (ZG Yang and LP Zhuang) independently assessed quality, and inconsistency was discussed with another reviewer-author (XR Chen) who acted as an arbiter.

### 2.4. Statistical Methods

Data were processed in accordance with the Cochrane Handbook [19]. Intervention effects were expressed as ORs and associated 95% confidence intervals (CIs) for dichotomous data and mean differences and 95% CIs for continuous data. Subgroup continuous data of each study were combined using the following formula [20]:

(1) SD=(((N1−1)SD12+(N2−1)SD22  +N1N2N1+N2  (M12+M22−2M1M2)) ×(N1+N2−1)−1)1/2,

where SD is the standard deviation, *N* is the sample size, and *M* is the mean.

Heterogeneity across studies was informally assessed by visually inspecting forest plots and formally estimated by Cochran's *Q* test in which chi-square distribution is used to make inferences regarding the null hypothesis of homogeneity (considered significant at *P* < 0.10). A rough guide to our interpretation of *I* ^2^ was listed as follows:

- 0% to 40% shows that heterogeneity may not be important;
- 30% to 60% corresponds to moderate heterogeneity;
- 50% to 90% exhibits substantial heterogeneity;
- 75% to 100% indicates considerable heterogeneity [19, 21].

If the eligibility of some studies in the meta-analysis was uncertain because of missing information, a sensitivity analysis was performed by conducting the meta-analysis twice: in the first meta-analysis, all of the studies were included; in the second meta-analysis, only those that were definitely eligible were included. A fixed-effects model was used initially for our meta-analyses; a random-effects model was then used in the presence of heterogeneity. Description analysis was performed when quantitative data could not be pooled. Review Manager version 5.1 software was used for data analysis.

## 3. Results

### 3.1. Study and Patient Characteristics

A total of 1,035 abstracts were reviewed; among these articles, 55 were retrieved, including 12 RCTs [8–12, 22–28] that are closely related to the current subject. However, two [22, 23] were excluded because these articles were basic research, two [24, 25] were excluded because these articles did not use placebo as a control, two [26, 27] were excluded because of duplication, and one [28] was excluded because of unavailable inclusion outcomes; hence, five RCTs [8–12] were selected on the basis of our inclusion criteria (Table 1).

A total of 222 and 167 patients were randomly treated with silymarin (or intravenous silibinin) and placebo, respectively. The baseline characteristics of patients included in this meta-analysis are described in Table 2.

### 3.2. Methodological Quality Assessment

**Figure 1:**
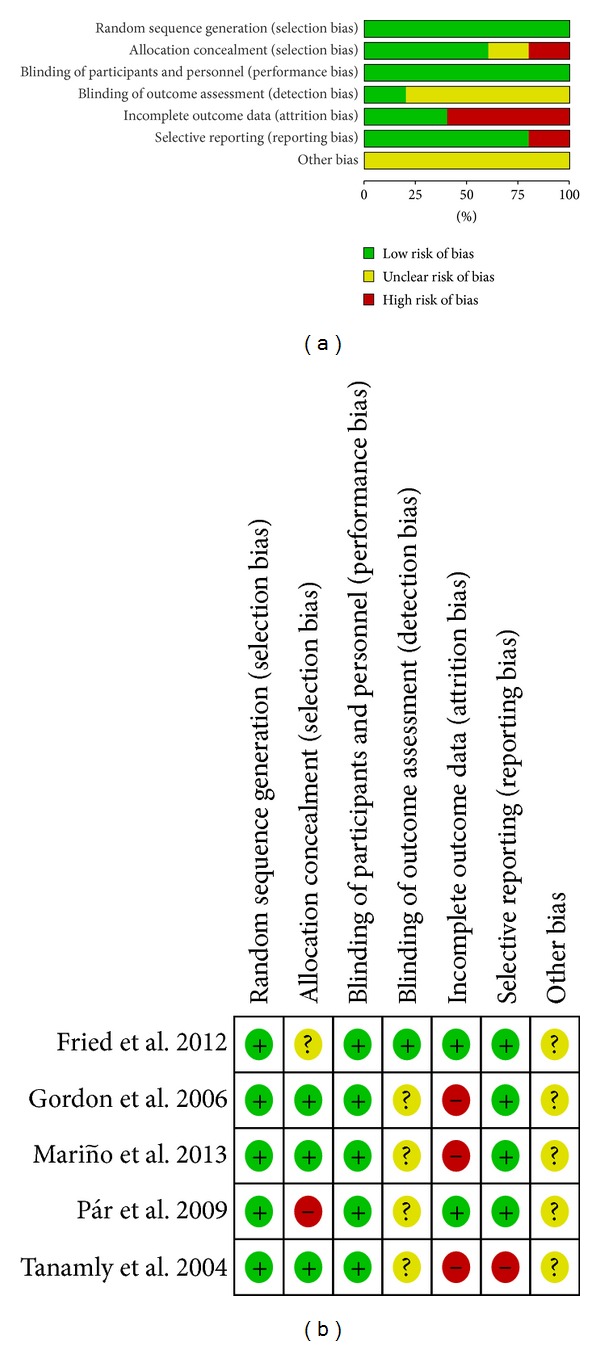
Risk of bias assessment. (a) Risk of bias graph: review authors' judgments about each risk of bias item presented as percentages across all included studies. (b) Risk of bias summary: review authors' judgments about each risk of bias item for each included study.

All of the studies included in this meta-analysis were described as randomized and double-blind. In two studies [8, 12], the method of randomization was not described, but randomization was adequate in other studies [9–11], which were considered as randomization number sequence [10, 11] and adaptive minimization-randomization scheme [9]. The statistical analyses in one study [10] were not based on intention-to-treat (ITT), and >20% of participants were lost to followup in the three studies [9, 10, 12]; these parameters were considered high risk in terms of incomplete outcome data. Only patients with HCV genotype 1 were included in the study of Pár et al. [8], which was considered high risk in terms of selection bias. In addition, the selection risk of a multiple-center study [11] was unclear, and detection bias was low. Selective reporting was found in another study [10] because this research failed to present the clinical data of participants in ITT analysis. Other potential biases were unclear in these trials (Figure 1).

### 3.3. Serum HCV RNA Titer

**Figure 2:**
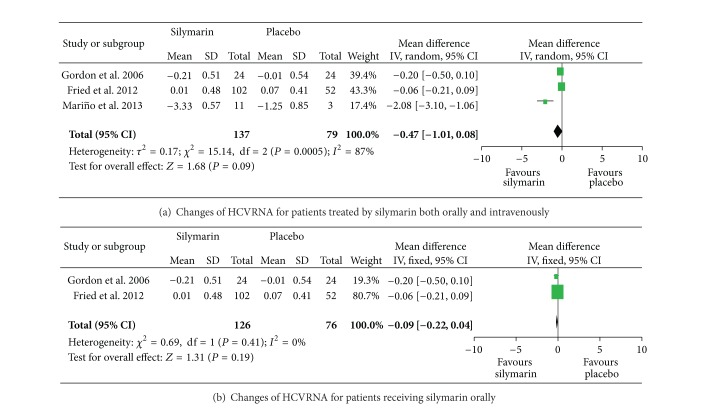
Changes of HCVRNA level.

Heterogeneity was significant among the included studies [9, 11, 12] in which changes in serum HCV RNA levels in patients treated with oral and intravenous silymarin and in patients treated with placebo were compared (*P* = 0.0005, *I* ^2^ = 87%). Thus, a random-effects model was applied; we found that serum HCV RNA relatively decreased in patients treated with silymarin compared with those administered with placebo, although no significance was found (*P* = 0.09, Figure 2(a)). However, no significant heterogeneity was found in a meta-analysis performed in patients treated with oral silymarin only, indicating that the changes in HCV RNA are similar to those of the two groups of silymarin and placebo (*P* = 0.19, Figure 2(b)). In the study of Tanamly et al. [10], HCV RNA persisted in 67/69 (97.1%) of the silymarin group and in 69/72 (95.8%) of the placebo group after 12 months of therapy. In the study of Pár et al. [8], the baseline HCV RNA level was higher in the placebo group than that in the silymarin group whereas the HCV RNA of patients who received placebo declined more significantly after 12 months of therapy than that in the silymarin group. The sustained virological response (SVR) in the placebo group was also higher than that in the silymarin group (43.8% and 18.8%, resp.). These contradictory findings of the trial reported by Pár et al. [8] may be related to randomization bias because patients in the silymarin group exhibited more negative predictors of response; for example, these patients were older with higher fibrosis score and showed more severe pretreatment baseline oxidative stress than those in the placebo group [8].

### 3.4. Serum ALT Level

**Figure 3:**
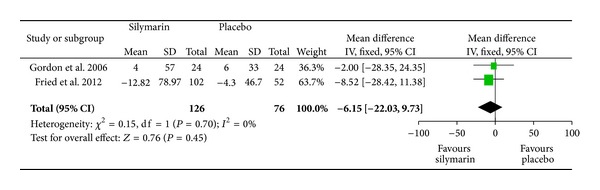
Changes of ALT level.

Considering that no significant heterogeneity was found among the included studies [9, 11] when changes in ALT levels of patients who received oral silymarin were compared with those who received placebo (*P* = 0.70, *I* ^2^ = 0%), we used a fixed-effects model and found that the effect of oral silymarin on ALT is not different from that of placebo (*P* = 0.45, Figure 3). No differences in ALT level changes were also found between responses to silymarin and placebo in the study reported by Tanamly et al. [10]. No adequate data of the effects of silymarin on ALT were available in the other two trials [8, 12].

### 3.5. Quality of Life, SF-36

**Figure 4:**
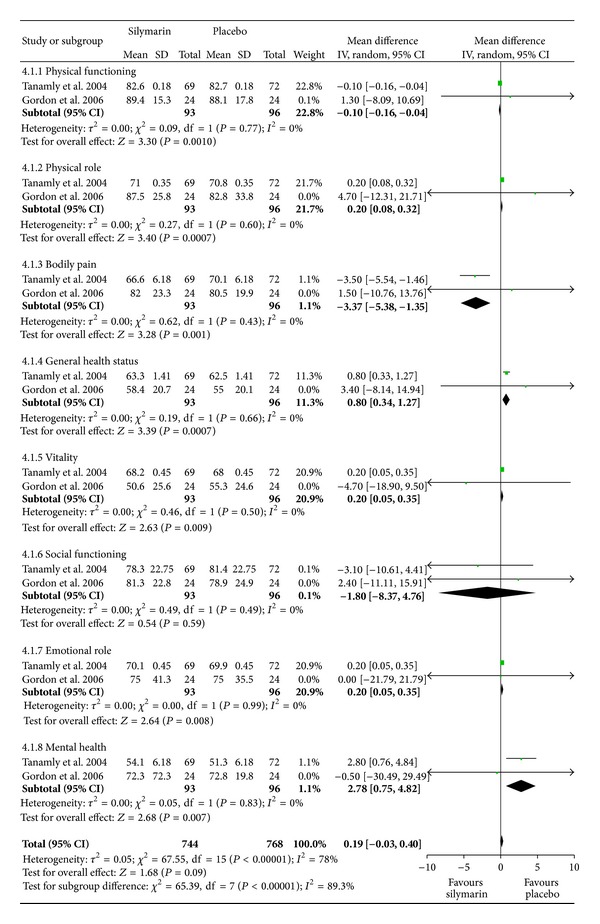
Quality of life by SF-36.

The SF-36 scores were not available in two studies [8, 12]. Heterogeneity was not found in all of the physical and mental variables of SF-36 [9, 10]. Silymarin treatment did not significantly affect social functioning score change compared with placebo (*P* = 0.59) (see 4.1.6 in Figure 4). However, the mean physical functioning and bodily pain scores decreased significantly during silymarin treatment (both *P* = 0.001) (see 4.1.1 and 4.1.3 in Figure 4). By contrast, physical role, general health status, vitality, emotional role, and mental health scores were significantly improved in the placebo group compared with those in the silymarin group (*P* = 0.0007, *P* = 0.0007, *P* = 0.009, *P* = 0.008, and *P* = 0.008, resp.) (see 4.1.2, 4.1.4, 4.1.5, 4.1.7, and 4.1.8 in Figure 4). Considering these results, we found that the effect on quality of life by SF-36 favored placebo, but no significant difference was found (*P* = 0.09) (Figure 4). In the study of Fried et al. [11], no significant changes were found in the physical or mental health components of quality-of-life scores (SF-36), in chronic liver disease health-related quality-of-life assessments (Chronic Liver Disease Questionnaire), or in depression scores (Center for Epidemiologic Studies Depression) in both silymarin and placebo groups.

### 3.6. Adverse Events

Abdominal tract discomfort/pain was the most frequently reported adverse event [9–12]. Headache was also commonly observed [9, 10, 12]. Dermatologic events were reported in two studies [9, 11]. Musculoskeletal pain and infection were found in the study of Fried et al. [11, 12]; fatigue was also observed [10, 12].

The types and frequencies of adverse events of participants did not differ across the two treatment periods of the studies reported by Gordon et al. [9] and Fried et al. [11]. Fever was frequently reported in the placebo group in the study of Tanamly et al. [10], but weekly incidence was sufficiently low (0.6%), indicating that this finding is negligible. Two patients withdrew from the study of Gordon et al. [9] because of adverse events. In summary, most of the adverse events were mild or not related to the study drug [9, 10, 12]. However, 12 serious adverse events were reported in the study of Fried et al. [11]: 1 in the placebo group and 11 in the silymarin group. Comparing placebo with silymarin, we found that the proportion of patients with at least one serious adverse event in the silymarin group (7/102) did not differ significantly from that in the placebo group (1/52; *P* = 0.27).

## 4. Discussion

The number of therapies against HCV has increased exponentially for several years. The efficacy of peginterferon alfa plus ribavirin administered for 48 weeks is correlated with HCV genotype, and SVR is only achieved in approximately 50% of HCV genotype 1 patients [29]. Direct-acting antivirals constitute a new stage in a recently approved HCV therapy that should improve SVR rates in both treatment-naive and treatment-experienced patients [30, 31]. With several limitations, including side effects, poor responses, and drug resistance of these therapies, alternative treatment strategies are urgently needed.

Silymarin has been used to treat many liver disorders, including acute and chronic viral hepatitis, alcoholic liver disease, hepatotoxicity, cirrhosis, and liver cancer [18, 23, 32–34]. In clinics, silymarin is widely used by subjects with HCV infection, although no strong evidence supports its usage [3]. Previous studies were criticized because of low methodological quality and small sample size. Fortunately, several strict-designed randomized double-blind controlled trials were published in the past decade [8–12]; however, conflicting results have been widely debated [15–18].

Previous studies observed that the main active mechanisms of silymarin involve inhibiting HCV entry and fusion, promoting HCV-induced oxidative stress, precluding HCV transmission, and blocking viral production [6, 35–38]. In our meta-analysis, a trend of beneficial effect on serum HCV RNA of silymarin was observed when both oral and intravenous silymarin were evaluated, although no significance was found. However, the advantage on HCV RNA levels partly disappeared when subjects treated with oral silymarin alone were considered. Hence, intravenous administration of silymarin may play an important role in inhibiting HCV replication. Ferenci et al. [13] reported that daily intravenous administration of soluble silibinin in previous peginterferon nonresponders inhibits HCV viral loads by three to four logs within one to two weeks. Silibinin is the major compound of silymarin and consists of two flavonolignans silybin A and silybin B; their water-soluble dihydrogen succinate forms inhibited HCV polymerase function with IC50s ranging from 75 *μ*M to 100 *μ*M [37]. In contrast to phenyl-benzopyrone structure, silymarin is relatively hydrophobic; thus, silymarin may act by incorporating into lipid membranes of both viruses and target cells or may at least display partition into lipid bilayers similar to other plant flavonoids. This partition would stabilize membranes as induced by silymarin, which would in turn become less prone to fusion [35, 39]. Therefore, treatment outcomes should vary with different routes of administration.

In our meta-analysis, the effects of silymarin on ALT levels of patients with HCV were similar to those of placebo; however, no beneficial aspect was found for silymarin. Interestingly, serum ALT is an arguably surrogate endpoint of monitoring treatment response in patients with HCV. Some reports have shown that decreases in serum ALT levels are highly correlated with improvement in hepatic necroinflammatory activity after interferon therapy is administered, although HCV RNA levels remain unchanged [40, 41]. Thus, improvement in hepatic histology or hepatic fibrosis would have been unlikely in the absence of any change in serum ALT level [11]. By contrast, Zarski et al. [42] believed that a poor correlation is observed between ALT levels and both hepatic necroinflammatory activity and fibrosis stage. Paired liver biopsies obtained before and after therapy should be assessed to determine treatment outcomes of silymarin in patients with chronic HCV.

The improvement of the quality of life of HCV-infected subjects is of great importance during treatment. In our study, silymarin could improve some symptoms, such as mean physical functioning and bodily pain, of patients with chronic hepatitis C. Comparatively, physical role, general health status, vitality, emotional role, and mental health status scores were significantly improved in the placebo group compared with those in the silymarin group. The improvements in the quality of life of silymarin and placebo recipients were impressive but relatively identical. Considering the safety of silymarin, we found that abdominal tract discomfort/pain was the most frequently reported adverse event. Headache, dermatologic events, musculoskeletal pain, infection, and fatigue were also commonly observed. All of these adverse events were mild and tolerable. Although the percentage of participants with serious adverse events was reported [11], the distribution of all of the adverse events was similar between treatment and placebo groups.

This study exhibits several limitations: (1) sample size was small with only 222 patients in the silymarin group and 167 in the placebo group; (2) treatment durations (28 days to 12 months) were different among the included studies, thereby requiring long-term therapy with silymarin; and (3) baseline characteristics, such as different virological responses of patients, different silymarin dosages, and different disease stages (including patients with HCV subjected to liver transplantation), were inconsistent.

Although silymarin is well tolerated in patients with chronic HCV, this meta-analysis suggested no evidence of salutary effects of oral silymarin on chronic HCV infection has been presented yet on the basis of intermediate endpoints (ALT and HCV RNA). However, intravenous administration of silymarin should be further investigated. Large-sample RCTs with more reliable endpoints, such as paired liver biopsies, should be performed in future studies to evaluate the effects and safety of silymarin for the treatment of patients with chronic HCV.

## Figures and Tables

**Table 1:** Baseline characteristics of included trials.

Study	Silymarin dose	Drug administration	Interferon therapy, *n* (%)	Intervention population	History of any milk thistle preparation use, *n* (%)	Silymarin treatment duration	Country	Study type
Tanamly et al. 2004 [10]	3 × 124.5 mg/day	Oral	NA	NA	NA	12 months	Egypt	RCT
Gordon et al. 2006 [9]	600 mg or 1200 mg/day	Oral	10 (41.7)	NA	NA	12 weeks	Australia	RCT
Pár et al. 2009 [8]	2 × 166 mg/day	Oral	32 (100)	Naïve	NA	3 months	Hungary	RCT
Fried et al. 2012 [11]	3 × 420 mg or 700 mg/day	Oral	None	Failed to interferon therapy	68 (44.2)	24 weeks	USA	RCT
Mariño et al. 2013 [12]	20 mg/kg/day	Intravenous	None	NVR = 11, naïve = 3	NA	28 days	Spain	RCT

NVR: nonvirologic response.

**Table 2:** Baseline characteristics of study participants.

Study	Intervention group	Total patients	Mean age (years)	Male, *n* (%)	HCV genotype (1/non-1/NA)	HCV RNA, mean ± SD/median (IQR)	ALT, mean ± SD/median (IQR) (U/L)	Albumin, g/L	Bilirubin, *μ*mol/L	BMI (kg/m^2^)
Tanamly et al. 2004 [10]	Silymarin	69	NA	NA	NA	NA	14 (20.6)^a^	NA	NA	NA
	Placebo	72	NA	NA	NA	NA	15 (21.1)^a^	NA	NA	NA

Gordon et al. 2006 [9]	Silymarin	24	43 ± 7	15 (62.5)	15/8/1	(4.7 ± 7.0) × 10^6^ copies/mL	100 ± 51	39 ± 3	10 ± 3	27 ± 5
	Placebo	24	43 ± 7	15 (62.5)	15/8/1	(4.7 ± 7.0) × 10^6^ copies/mL	100 ± 51	39 ± 3	10 ± 3	27 ± 5

Pár et al. 2009 [8]	Silymarin	16	51.6 ± 6.3	8 (50)	16/0/0	641 ± 178 kIU/mL	141.3 ± 85.5	NA	NA	NA
	Placebo	16	46.8 ± 9.9	5 (31)	16/0/0	740 ± 227 kIU/mL	108.7 ± 52.7	NA	NA	NA

Fried et al. 2012 [11]	Silymarin	102	54 (48–58)	79 (77.5)	95/7/0	6.1 (5.7–6.5)^b^; 6.3 (5.8–6.6)^c^ log_10_⁡IU/mL	109.5 (83–158)^b^; 104.5 (83.5–151)^c^	41 (39–44)	0.8 (0.6–1.0)	28.5 (26–32.4)^b^; 30.2 (28.1–32.9)^c^
	Placebo	52	56 (51.5–59.5)	41 (78.9)	44/6/2	6.4 (5.9–6.7) log_10_⁡IU/mL	106 (83–136)	43 (39–45)	0.9 (0.6–1.1)	29.1 (26.5–32.7)

Mariño et al. 2013 [12]	Silibinin	11	57.2 ± 9.3	6 (54.5)	9/2/0	(5.17 ± 0.76) log_10_⁡IU/mL	NA	NA	NA	NA
	Placebo	3	52 (41–62)	3 (100)	3/0/0	4.94 (4.46–5.95) log_10_⁡IU/mL	NA	NA	NA	NA

^a^Presented by *n* (%) of participants with ALT abnormal; ^b^data of participants receiving 420 mg per time three times a day; ^c^data of participants receiving 700 mg per time three times a day.
